# Mental health and COVID-19 in university students: a qualitative study comparing Italy and the UK

**DOI:** 10.1192/j.eurpsy.2023.492

**Published:** 2023-07-19

**Authors:** I. Riboldi, C. A. Capogrosso, S. Piacenti, A. Calabrese, S. Lucini Paioni, F. Bartoli, J. Armes, C. Crocamo, C. Taylor, G. Carrà

**Affiliations:** ^1^Department of Medicine and Surgery, University of Milano-Bicocca, Monza, Italy; ^2^Faculty of Health and Medical Sciences, School of Health and Sciences, University of Surrey, Stag Hill, Guildford; ^3^Division of Psychiatry, University College London, London, United Kingdom

## Abstract

**Introduction:**

The worldwide spread of the COVID-19 pandemic affected all major sectors, including higher education. The measures to contain this deadly disease led to the closure of universities across the globe, introducing several changes in students’ academic and social experience. During the last two years, self-isolation together with the difficulties linked to online teaching and learning, have amplified psychological burden and mental health vulnerability of students.

**Objectives:**

We aimed to explore in depth students’ feelings and perspectives regarding the impact of the COVID-19 on their mental health and to compare these data among students from Italy and the UK.

**Methods:**

Data were resulting from the qualitative arm of “the CAMPUS study”, a large ongoing project to longitudinally assess the mental health of university students enrolled at the University of Milano-Bicocca (Unimib, Italy) and the University of Surrey (UoS, Guildford, UK). We conducted in-depth interviews through the Microsoft Teams online platform between September 2021 and April 2022, and thematically analysed the transcripts.

**Results:**

A total of 33 students (15 for Unimib and 18 for UoS), with a wide range of sociodemographic characteristics, were interviewed. Four themes were identified: i) impact of COVID-19 on students’ mental health; ii) causes of poor mental health; iii) most vulnerable subgroups; vi) coping strategies.

Anxiety symptoms, social anxiety, and stress were frequently reported as negative effects of the pandemic, while the main sources of poor mental health were identified in loneliness, exceeding time online, unhealthy management of space and time, bad organization/communication with university, low motivation and uncertainty about the future. Freshers, international or off-campus students, as well as both extremely extroverted and introverted subjects, represented the most vulnerable populations, because of their extensive exposure to loneliness. Among coping strategies, the opportunity to take time for yourself, family support, and mental health support were common in the sample.

Some differences were found comparing students from Italy and the UK. While at Unimib the impact of COVID-19 on mental health was mainly described in relation to academic worries and the inadequate organization of the university system, UoS students, familiar to the conviviality of campus life, explained these effects as a result of the drastic loss of social connectedness.

**Conclusions:**

The current study highlights the key role of mental health support for university students, mainly during crisis times, and calls for measures to improve communication between students and the educational institution, as well as to encourage social connectedness.

**Disclosure of Interest:**

None Declared

